# Exploring formal and informal learning opportunities during morning report: a qualitative study

**DOI:** 10.1186/s12909-024-05151-5

**Published:** 2024-02-23

**Authors:** Jane Ege Møller, Mads Skipper, Lone Sunde, Anita Sørensen, Thomas Balslev, Bente Vigh Malling

**Affiliations:** 1 Department of Clinical Medicine, Aarhus University, Olof Palmes Allé 15, 8200 Aarhus N, Denmark; 2 Educational Region North, Viborg, Denmark; 3 Department of Clinical Genetics, Aalborg University Hospital, Aalborg, Denmark; 4 Bispebjerg & Frederiksberg Hospital, Capital Region of Denmark, Aalborg, Denmark; 5 Viborg Regional Hospital, Central Denmark Region, Viborg, Denmark

**Keywords:** Workplace learning, Teaching styles and format, Informal and formal learning opportunities, Postgraduate medical education, Clinical education, Morning report, Qualitative research

## Abstract

**Background:**

Morning reports are an essential component of physicians’ daily work. Attending morning reports is prioritized by junior doctors as it provides them with an opportunity to learn diagnostic reasoning through discussion of cases. While teaching formats during morning reports have previously been reported, an in-depth analysis of what learning opportunities exist, e.g., how teaching is enacted during morning reports, is lacking. This qualitative study explores learning opportunities during morning reports.

**Methods:**

We used an explorative design based on video-recordings of 23 morning reports from two surgical departments, an internal medicine department and an emergency department. We used thematic analysis combined with and inspired by Eraut’s theoretical framework of workplace learning.

**Results:**

Both formal and informal learning opportunities were identified. Formal learning opportunities had the character of planned teaching activities, and we identified four themes: (1) modes of teaching, (2) structure, (3) presenter role, and (4) participant involvement. Informal learning, on the other hand, was often implicit and reactive, while deliberate learning opportunities were rare. The data showed many missed opportunities for learning.

**Conclusion:**

Both formal and informal learning opportunities are present during morning reports. However, a prevalent focus on medical topics exists, leaving other important aspects of the medical role under-discussed. Pedagogical methods could be employed more optimally, and harnessing the potential of missed opportunities should be encouraged.

## Background

Medical specialist training consists primarily of workplace-based education [1–6]. Globally, the morning report, where all physicians in a department meet, is an essential aspect of the daily work in most specialties [7–10]. Morning reports have evolved from centering on patient handovers and ensuring the quality of patient treatment to including regular educational components [9, 11–14]. Junior doctors appreciate the learning opportunities at morning reports and prioritize participating in these meetings [14, 15]. Morning reports have become an important way of improving specialist training [8, 15–17], especially as case-based discussions supplement lecture-based educational activities [12, 14, 18–20]. Knowledge about the teaching activities in morning reports is therefore key to understanding how to ensure ideal learning opportunities for junior doctors.

Studies have shown that morning reports improve both clinical reasoning and learning if all physicians participate in the discussions [19, 20]. However, measuring learning outcomes is challenging since morning reports have heterogeneous aims, forms and settings [9]. Recent studies have used quantitative methods to investigate teaching and learning in morning reports. In their study, Redinger et al. investigated participation at internal medicine attendings [21]. Heppe et al. [13] found that teaching during morning reports was typically prepared, moderated and presented by a junior doctor using a digital presentation, and that it tended to comprise a single case and include discussions about possible diagnoses. While such studies provide valuable overviews, e.g., of formats used, they do not provide in-depth knowledge of how teaching activities took place. Such knowledge would be valuable for mapping learning practices and identifying ways of strengthening and supporting learning in morning reports.

Since social, cultural and contextual factors influence the ways in which morning reports are conducted and play an important role in junior doctors’ professional development and socialization into the medical community [9, 11, 14, 22–24], a qualitative approach would provide a useful supplement to quantitative overviews.

This study provides such a qualitative approach. Our research question was: What opportunities for learning exist during morning reports?

## Methods

This study is part of a larger project that examines two dimensions of the morning report: a socio-cultural dimension and an explicit teaching and learning dimension. The socio-cultural dimension has been described in a previous study [24]. As the present research aimed to explore what opportunities for learning exist during morning reports, we used a qualitative and explorative design based on video-recordings of morning reports in four different hospital departments in Denmark.

### Setting

In Denmark, the morning report is an everyday routine in almost all departments and specialties and is more comprehensive than practised in other countries. Described as a “morning meeting” or ‘morgenkonference’ in Danish, this mono-professional meeting occurs at the outset of the day for all doctors in most Danish hospital departments and typically lasts between 15 and 45 min, the latter once or twice per week. Participation is expected from all medical staff on duty, i.e., doctors, residents and students. Alongside case-based learning and brief presentations, the morning report comprises various elements such as handovers, planning daily work, discussions about treatment for patients and planned educational sessions. As such, the morning report in Denmark has a broader scope than in North America where morning reports primarily relate to internal medicine and are exclusively based on case-based educational sessions [14, 25, 26].

### Data collection

We followed the principle of variation by inviting different specialties, and wished to include medical, surgical, psychiatric and emergency departments. We invited seven departments, four of whom agreed to participate. The psychiatric department declined participation, so we incorporated an element of convenience sampling by leaving this perspective out. Knowing that specialties and departments vary in relation to size, cultural characteristics and composition as regards educational level, we made video-recordings in an emergency department, an internal medicine department and two surgical departments (see Table 1 for an overview). Video-recordings were chosen as the method to capture the interactions because it allows researchers to capture simultaneous complex interactions and activities in their natural settings over time [27]. The first author and two research assistants undertook the video-recordings using video cameras for the recording. These were managed by the person in charge of recording and were visible to the participants at all times. The recordings were first stored in the camera memory card. Later they were copied to a secure database. The typical report duration was 15–20 min, varying from approximately 7 to 45 min. The latter was unusual– occurring, e.g., when a department had allocated teaching time for a guest lecturer. During the analysis, central passages representing teaching activities and learning opportunities were selected. These were transcribed into field notes, and verbatim excerpts of dialogue were included.

### Ethics

The project followed the ethical principles for medical research as defined in the WMA Helsinki Declaration [28]. The project group contacted Heads of Department (HDs) and informed them about the project. The HDs informed their employees and ensured willingness to participate. The first author then presented oral and written information about the project to all of the participants, all of whom provided written consent. All transcripts were anonymized. As the patients only participated indirectly in the research project, we were exempted from obtaining written consent from them. The study was exempted from ethics approval according to the Act on Research Ethics Review of Health Research Projects. The Danish Data Protection Agency approved the study (Journal Nr. 2016-051-000001).

**Table 1:** Overview of data collection, participating departments and doctors

	Consultants, specialists	Junior doctors	Total number of doctors employed	Approximate number of participants in each morning report	Video-recordings
Emergency	10	16	26	10–15	6
Surgery 1	22	20	42	20–35	5
Surgery 2	18	8	26	15–20	6
Internal medicine	26	34	60	35–45	6
Total	76	78	154	-	23

### Analysis

The authors performing the analysis were a cross-disciplinary group of researchers from medicine, medical education, anthropology and communication. The analysis had an initial explorative phase, where we used thematic analysis as described by Braun and Clarke [29, 30] to identify initial codes and themes (steps 1–3 in Table 2). Then, we defined the final themes drawing on the lens of Eraut’s theoretical framework for learning [1] (steps 4–5 in Table 2; see Table 2 for a detailed description.)

Eraut described how workplace learning takes place at three interconnected levels: the individual, the team and the organisational level [31]. Learning depends on the learner’s capability and the context in which learning takes place, and it includes both working and learning conditions. Eraut described how learning can be the result of either formal or informal learning activities. Formal learning activities involve components such as a prescribed learning framework, an organised teaching/learning event, the presence of a designated teacher, the award of a qualification and external specification of outcomes [1]. As we analysed the learning opportunities in the workplace during morning reports, the last two components did not play a significant role. Informal learning is defined as a continuum and includes (1) implicit learning, which implicitly links past memories with current experience, (2) reactive learning, which involves a brief spontaneous reaction to past episodes and events, recognizing the potential for future learning, and (3) deliberative learning, which involves the discussion and review of past actions and events, combining them with planning and rehearsing future activities [1].

Eraut’s description of learning in the workplace was chosen as the analytical lens [1, 3, 4] because it embraces and conceptualizes both formal and informal learning, and thus enables us to grasp different types of learning opportunities. However, while Eraut’s framework focuses on broad aspects of workplace learning, we narrowed the focus to potential learning opportunities. We thus selected part of Eraut’s framework in the analysis. By using the term ‘learning opportunity’, we emphasise that our aim was to capture various activities that could *give rise to learning opportunities*, as opposed to *actual formal and informal learning*. The latter would necessarily involve another research design, e.g., based on interviews.

All participants were anonymized using the following abbreviations: Junior doctor = JD; medical specialists and consultants = SP; Head of Department = HD. The departments were anonymized using the following acronyms: Internal medicine = IM; Surgical departments = S1, S2, and Emergency department = EM, followed by a number referring to the video recording– e.g., IM-17. In the examples, we have used pseudonyms for names to ensure confidentiality. Moreover, some topics discussed have been hidden, e.g., by replacing them with “XX” for anonymity concerns.

**Table 2:** Overview of data analysis

Step	Activity
1	All authors watched and discussed six randomly selected video-recorded morning reports while sitting together. During each video-recording, each author wrote down their individual reflections. Notes were shared and preliminary themes were identified. Two analytical dimensions, a socio-cultural dimension and an explicit teaching and learning dimension, were identified. The socio-cultural dimension has been described in a previous study (24).
2	The first and last author then re-watched all 23 recordings individually and made detailed descriptive notes on paper about the interactions, focusing on teaching activities and learning opportunities.
3	The notes were compared, and initial codes were identified using an inductive and explorative approach. Selected video excerpts were watched several times to review the codes and identify initial themes.
4	Further analysis was then undertaken using Eraut’s theoretical framework as a lens and defining final themes. Selected passages were transcribed verbatim.
5	Analysis concluded with a final check and adjustment of themes.

## Results

We present the analysis using the two modalities from Eraut’s framework: formal learning opportunities and informal learning opportunities. For each of the modalities, several themes were identified. Table 3 provides an overview of the modalities and themes observed.

**Table 3:** Overview of data analysis: modalities, themes and code, examples/explanation

Modality	Theme	Codes, example/explanation
Formal learning opportunities	Modes of teaching	Extensive presentation/lecture (30–45 min) E.g., internal or external presenters invited to speak, topic decided by presenter or presenter asked to speak on specific topic
		Teaching of the day (6–20 min) E.g., junior and senior doctors present, topic decided by presenter relevant for specialty or department, can relate to actual patient or season
		Case of the day (5–15 min) Often junior doctors, patient case decided by presenter, can relate to actual patient in department
	Structure	Classic case presentation E.g., extensive presentation or teaching of the day, topic decided by presenter
		Scientific presentation (IMRaD) E.g., extensive presentation or teaching of the day, topic decided by presenter
	Presenter role	Opening remark E.g., humor, own credentials, apology
		Power relation E.g., senior or junior doctor, expert or learner
	Participant involvement	Questioning Asking audience questions (open and closed)
		Story, quiz Presentation like a ”crime story” where audience helps story proceed to solution by asking or answering questions
		Discussion, elaboration Inviting audience to elaborate further or open discussion on topic presented
		Attempted involvement E.g., presenter invites audience to ask questions, but does not really give sufficient time or space for questions during or after presentation
		Attempted case structure E.g., many patient cases presented during patient handover but no time for questions
Informal learning opportunities	Implicit teaching	Role modelling E.g., rich opportunity to see how others behave and react during morning conferences
		Illness script formation Both case presentations and patient handovers provide opportunity to build on prior knowledge of specific diseases. Helps audience create patterns of diseases
		Manager role How to conduct a morning conference, how to deliver morning report
		Professional role (ethical dilemmas) When to tell patient and relatives that further treatment is pointless
		Communicator How to break bad news, how to respond to non-compliant patients
		Collaborator Collaboration with other health care staff, collaboration with other departments
	Reactive situations	Seizing the opportunity Elaborate on a case presentation or patient handover by adding what could otherwise have been done or what would have been better to do, adding own experience with similar patient
	Deliberate teaching opportunities	Announcements Announcing future planned educational activities, lectures, workshops or conferences
		Presenting or consulting patients Going back to patients discussed earlier or providing information on similar patients in relation to a case presentation or patient handover
	Missed opportunities	Revisiting cases Following up on patients to show course of diseases
		Communication/ethical dilemmas Handling non-compliant patient, handling angry relatives, dealing with ethical dilemmas like when and how to deliver bad news
		Manager role How to manage patient handover, how to foster good collaboration with other departments, how to solve managerial problems between departments, how to do data registration and why
		Clinical experience Responding to specific clinical symptoms by suggesting junior doctors go and see that patient

### Formal learning opportunities

Formal learning opportunities were identified as planned teaching activities where time was allocated to teaching and someone was appointed or invited to present. Four themes were identified: (1) modes of teaching, (2) structure, (3) presenter role, and (4) participant involvement. These are described in more detail below.

#### Modes of teaching

Three modes of teaching were identified: the extensive/long presentation, the teaching of the day, and the case of the day.

Extensive presentations/lectures typically occurred in the departments once a week and involved both internal and external presenters. The topics were predominantly biomedical, and included diseases, symptoms, drugs or biomedical research; the only exception was a presentation from an external presenter on the topic ‘Feedback’.

The teaching of the day was more frequently observed; it was shorter and mostly performed by a junior doctor. The topics were all biomedical and presented a diagnosis and treatment. In one department, someone was occasionally appointed to provide feedback to the presenter, but in most cases the feedback was restricted to ‘thank you for the presentation’.

The case of the day was just as short as the teaching of the day, but it related to a specific patient recently admitted in the department, whom the presenter found to be of common/general interest.

Thus, overall, formal teaching activities primarily related to the ‘medical expert’ aspect of patient care and only a few examples incorporated more social or ethical aspects.

#### Structure

In most teaching, the structure followed the traditional case presentation structure of ‘definition, epidemiology, symptoms, diagnosis and treatment’ [32, 33] or the classical scientific presentation IMRaD: introduction, methods, results and discussion/conclusion [34].

A few presenters related topics to the department’s current work or stated the relevance of their topic, as witnessed in the following ‘teaching of the day’ excerpt [EM-21]:

*I prepared a talk about ‘influenza’, because it is the flu-season and we are currently admitting many patients with influenza in the department.* [JD]

In these cases, linking the teaching topics to everyday clinical work created coherence between theoretical and evidence-based knowledge and practical clinical examples. However, these were rare, and more often a reason for presenting a specific topic was absent, as witnessed in the following ‘teaching of the day’ example [S1-1]:

*I have prepared a presentation of a scientific randomized study published some years ago. It shows the difference between using the XX vacuum system one or several days after the operation, respectively.* [JD]

The JD presents the study using the IMRaD structure.

*What kind of vacuum system did they use in the study?* [SP]

The JD answers.

*We stopped using that kind of vacuum system years ago, so…* [SP].

This example illustrated how, even if the topic seemed relevant, a presentation might not relate to actual practices in the department. This was a dominant pattern, and it resulted in the teaching occurring as an activity that did not clearly relate to the clinical work.

#### The role of the presenter

Often, the presenters in the formal teaching were internal, and presenting for colleagues seemed to involve a navigation of roles. Thus, internal presenters commonly started their presentation with comments like: ‘This might be a bit too basic…’ or ‘You probably already know this…’. Such opening remarks, which suggested that the audience’s level of knowledge about the content presented exceeded the presenter’s own, seemed to modify the presenter’s position. This kind of modification was witnessed across junior-senior levels.

Another type of opening involved humor [IM-16]:

*Actually, I promised you, Peter, that I would talk about XX, but then I realized that would be too nerdy. So Peter, you can go back to sleep. Because now I will present a totally different topic…* [SP].

Such performances indicated that the relationship between the audience and presenter was complex due to the workplace context where one presented in front of colleagues. In addition, it highlights that formal teaching in the workplace, where one presents for a group with heterogeneous competencies and experience levels, involves addressing the relationship between oneself as a presenter and the group more so than, e.g., would be the case in lectures in medical school.

#### Participant involvement

The most common participant involvement observed was inviting the audience to ‘ask questions during or at the end of the presentation’. Occasionally, no participant involvement at all was seen, but a few times the teaching maximized involvement, for example, by stopping the story and inviting colleagues into a clinical reasoning exercise that allowed them to ask questions and suggest a diagnosis [S2-10]:

*Today’s case is a 33-year-old male re-admitted for pain in the right side of his groin. Any suggestions for a diagnosis?* [JD1]

*How were the blood tests?* [SP]

*Other symptoms?* [JD2]

*Fever, however, inconstant. Sometimes very high, sometimes no fever– further suggestions on the diagnosis or what to do?* [JD1]

*What about appendicitis?* [JD3]

*Good point - but the clinical signs were inconsistent. Other suggestions?* [JD1]

Several diagnoses were suggested by both JDs and SPs, and then JD1 continued:

*Do you want to know the whole story? We finally figured out that he was suffering from tuberculosis.* [JD1]

Cases of ‘attempted participant involvement’ were frequently observed, such as when the presenter asked a question but continued before letting the audience answer. In the following, the presenter had given the background for a specific disease and showed a slide with possible treatments [IM-16]:

*I will give you a quiz now. How would you treat him/her? I will only give you a few seconds to answer this question.* [SP]

Immediately, before anyone else had time to answer, he answered the question himself, shutting down the possibilities for the audience to answer. The pedagogical opportunities were thus not used to their full potential.

Another type of attempted participant involvement took the form of ‘examining the audience’ either by asking a named individual and somehow mimicking a traditional examination where a senior staff member questions individual students or trainees. This was evident, for example, when a senior doctor and external presenter, after describing a case, asked everyone in the audience to answer a question, but moved quickly on to asking a named senior doctor directly [IM-13]: *What would your spontaneous diagnosis be?… Hanna, what is this? What is the matter with this patient?* [SP]. This example illustrates that participant involvement was not fully achieved because the focus was quickly directed at one member of the audience. Thus, such attempted participant involvement had several modes and indicated that pedagogical ideals for such teaching existed, though were not always fully exploited.

### Informal learning opportunities

Under the modality of informal learning opportunities, four themes inspired by Erault’s conceptualization were identified, namely, implicit learning opportunities, reactive situations, deliberative learning and missed opportunities.

#### Implicit learning opportunities

Implicit learning opportunities related primarily to daily work-planning, diagnostic reasoning, and cooperation with other stakeholders.

In all departments, the daily work schedule was confirmed or altered if needed during the morning report. The way in which the HD or the specialist in his/her place relocated staff to ensure that all activities were covered showed specialists and trainees how to manage a staff shortage, serving as implicit role modelling.

Describing the patients who were admitted during the night shift was another potential learning opportunity. Although many patients were only briefly mentioned using the typical case-presentation or elements of it, the story of how the doctor on call diagnosed and treated the patient offered an implicit opportunity to strengthen the development of illness scripts in the audience (Schmidt et al. 2007).

The material showed variations in how the patients were talked about. In most cases, the patient was mentioned by his/her full name as in the following [S2-7]:

*Gitte Jensen is back, again with respiratory problems…* [JD].

However, a few patients were depersonalized and reported as the ‘*58-year old male appendicitis, still in the recovery room,’* suggesting a more medical focus.

Implicit learning opportunities also related to cooperation, which was typically observed in ‘talk about other colleagues and stakeholders’. Examples were often negatively loaded and concerned something others had done wrong, e.g., deviating from agreed procedures as in this example [S2-11]:

*I have been informed that the patient who had XX yesterday has already been referred to the ward from the recovery room. This is not in accordance with the agreement we have with the anesthesiologists. It is unacceptable.* [SP]

On other occasions, the cultures of other groups of doctors, specialists or specialties were mentioned regarding how diagnosis and treatment were not in accordance with the way ‘we’ think it should be done [IM-16]:

*I guess it has to do with the way they* [the other specialty/department] *diagnose these kinds of patients. They use different tests than we do. And once the patient has got the diagnosis, he/she is referred to us to complete the treatment.* [SP]

Such occasions provided potential learning opportunities, because participants in the morning report were able to hear these modes of discourse as ways of ‘how to talk about patients’ or ‘how to address cooperation’.

#### Reactive learning opportunities

Reactive learning opportunities were spontaneous opportunities for learning that were picked up and addressed as they occurred. Although rare, random and not incorporated into everyday morning report practice, such examples were present in the material, such as the following example [EM-18].

*When you receive a patient with a seizure, remember that the patient must be informed about driving prohibition.* [SP]

Mostly senior doctors seized such learning opportunities by explicating what type of learning should be drawn. Such teachable moments often featured biomedical issues and legal restrictions, as in the last example, or managerial issues as illustrated in the next example:

During announcements, a secretary in the department urged all doctors to remember to register the patients in the databases. The HD seized the opportunity to explain the reason for registration in databases [EM-23]:

*Registering the patients and the medicine correctly is a very important part of quality control.* [HD]

*What is it used for?* [JD1]

*Is it for the patient’s sake or ours?* [JD2]

*Everything we do is for the patient.* [HD]

*Well, how do we do it?* [JD1]

The HD seized the opportunity, opening up the registration system and giving several examples of how to register correctly. This exemplifies how to capture the possible learning in an episode, recognizing the potential for future learning by making it a teachable moment.

#### Deliberative learning opportunities

Deliberative learning opportunities were less frequent, and they involved announcements like the following [IM-14]:

*I just want to mention that there is bedside teaching today at… let’s say 11.45.* [JD]

They could also involve reviews of past actions and events like the next example where a senior consultant referred to a patient from the previous day [S2-8]:

*Yesterday, we discussed a patient with XX that was treated correctly with a drainage. After some hours of observation, we decided to perform a more extensive operation, due to the patient’s symptoms. You can learn two things from this. Not that the patient did not receive the right treatment in the first place– he did– but you could have decided to do the extensive operation in the first place, which probably would have spared the patient the second operation.* [SP]

In this way, he corrected the treatment, without blaming the junior colleagues involved, and created space for reflection on a previous treatment and possible future optimizations.

### Missed opportunities

The last theme was missed opportunities. These included situations where dialogue about a patient, treatment or organisational issue was not unfolded, and an opportunity for creating an explicit or reactive teaching situation was not used. Missed opportunities mostly related to non-biomedical aspects such as ethical dilemmas, communication, and patient compliance.

For example, a junior doctor reported that an older woman suffering from cancer was referred from another hospital where she had had an extensive operation [S2-8]:

*She is going to die. It cannot be cured– no matter what we do.* [SP1, S2-19]

*Are the relatives informed?* [SP2]

*Yes, they are informed that the condition is critical.* [JD]

*But what about the prognosis?* [SP2]

*Do they know that this cancer is incurable?* [SP1]

Since these questions remained unanswered, it is difficult to deduce from this exchange what had been discussed with the patient and the relatives, and how the JD dealt with breaking bad news. It thus shows a situation that could have been used to discuss how to communicate with patients and relatives in case of incurable disease.

The following example deals with cooperation with other departments. While talking about the day’s program in the operating theatre, a consultant announced three small procedures, all decided by another department. This initiated a rather heated discussion about how a different department could schedule patients without concrete agreements about date and time. One consultant said [S2-9]:

*How can they make a booking in our system that we cannot see?* [SP1]

*It is not only a matter of booking– it is also about agreement between more than the two departments directly involved.* [HD]

*How can we change that– and make it more functional?* [SP2]

The HD chose to close the discussion by taking on the problem him/herself. The HD might have decided that this was not the time nor the place nor the right audience to take up a discussion like this. However, it could been have used to discuss how to communicate about patients between departments, how to make proper referrals, how to give notice as a service and even how to reach agreement between departments as a learning situation regarding cooperation.

Another kind of missed opportunity is reflected in the following example regarding non-compliance [S1-4]:

*Yesterday I operated Ian again– you all know Ian? Ian is the only patient I know who can break sutures time and again.* [SP1]

Everybody nods. SP1 turns to the students and describes Ian’s case, ending with:

*No matter what I tell him not to do– he will do it. He does not listen to any guidelines or precautions at all.* [SP1]

In this situation, how to deal with frustration about such patients remained unaddressed and nobody discussed how to communicate with non-compliant patients in the future.

The last example of a missed opportunity involved overlooking an opportunity to present a clinical phenomenon [S2-10]:

*Yesterday, we received a patient with subcutaneous emphysema. I have never seen this before– the patient is well this morning although it is still present.* [JD]

This case presentation could have been followed by a suggestion to students and other junior doctors to go and see the patient in order to gain experience, but this was overlooked.

## Discussion

This study has shown that morning reports provide learning opportunities in various ways. Using the definitions provided by Eraut, both formal and informal learning opportunities were present. The learning opportunities were presented using a variety of pedagogical tools and included diverse themes from medical expertise to social, ethical and organisational themes.

The formal teaching activities mainly took the form of a presentation of a medical problem, illness or case following the case presentation script [32, 33], or followed the IMRaD structure of scientific presentation [34]. These two ways of presenting resemble the ways in which doctors are trained in the ‘language of medical case histories’ and how to give a scientific presentation [33, 35]. These modes are central to the communication between doctors about patients, e.g., in patient handovers [22]. However, it is questionable whether they are effective in formal educational activities, like the presentation or case of the day. Here, a more Socratic questioning style and presentation of a case as a ‘crime story’ could invite the audience to take part more actively in the discussion and stimulate reflection around diagnostic reasoning [19]. Only a few examples of this exemplary way of presenting a case were seen in our data, pointing to further potential for teaching and learning during morning reports from activities that already take place.

Although the presenters seemed aware of learner-activating strategies, e.g., questions or cases, their pedagogical potential was not fully utilized as shown when they did not allow the audience time to answer or when they used inquisitorial-style questions. Future studies, for example involving interviews, could provide insights into the difficulties in using more learner-centered teaching styles.

It is interesting that presenters often opened their presentation with an apologetic remark. This could reflect that teaching one’s colleagues is not necessarily an easy task. As a presenter, it is necessary to balance between being too trivial (everyone knows the topic) and being too specialized (only a few understand) to comply with adult learning theories [5]. As such, teachers in morning reports seem to be on a ‘mission impossible’, unless they actively use the expertise in the room by truly involving participants.

The formal teaching activities primarily dealt with the ‘medical expert’ part of patient care. While only a few attempts to incorporate more social or ethical views of a patient or an illness were seen, these were far more visible in the non-formal teaching moments. This resonates with other studies that found that topics relating to medical expertise dominate in morning reports, while other dimensions like communication or ethics are less included [13, 36]. It thus seems a part of the ‘hidden curriculum’ and a way of reproducing biomedical dominance in teaching activities. This suggests that more explicit attention should be given to teaching other topics if a broader view on the role as a doctor, e.g., as seen in the CanMEDS framework [37] is to be an integrated part of the morning report.

Informal learning opportunities were richly represented in the data, but often passed unnoticed or at least were not discussed, and thus ended up as missed opportunities. Additionally, informal learning opportunities featured a variety of themes besides medical expertise and, if grasped, might have stimulated reflections on ethical, social and organisational aspects of patients and patient care. The importance of supporting the development of doctors’ professional identity by training a variety of roles besides the medical expert role have been stressed by other authors [37, 38].

Unlike formal teaching activities, informal learning opportunities often related to daily clinical work and actual patients. When these opportunities were seized, they provided significant situations where here-and-now problems were discussed. It is beyond the scope of this study to explain why these opportunities were not seized to a greater degree. Further studies including feedback to the departments might account for such missed opportunities and could enhance awareness of learning opportunities during morning reports.

Implicit learning opportunities like handling the working plan in the case of staff shortages or other administrative duties are not articulated as learning opportunities. However, by listening to the various ways in which these administrative duties were managed, all participants can learn what to do in the future. This could be an example of the “hidden curriculum” and how tacit knowledge is produced [1] or how doctors implicitly add meaning to what are explicitly interpreted as routine activities [1]. No one explicitly addressed how to learn to plan a day’s work, but it was more or less done in the same way in all departments, and thus transfers unnoticed (in the form of implicit learning) from one generation to the next and repeats itself [1, 39], possibly through role-modelling, or as part of the hidden curriculum [1, 4, 40].

Our study shows that much of what happens during morning reports is not labelled ‘education’ and might not be recognized as such, even though education is one of the many reasons for having morning reports [11, 12, 16, 22, 23]. Eraut’s framework provided a valuable theoretical lens to grasp different types of teaching activities and learning opportunities that might be used or used better to make the most of the little time doctors spend together.

Our study has limitations. Although observations are well-suited to describe possible teaching activities and opportunities, it is not possible to say whether learning actually took place. This is a methodological limitation, which could be redressed through interviews, though this lay outside the scope of the present study. Methodologically, the use of video observations raises the question of participant reactivity, i.e., if the method resulted in changes in participants’ behaviors. However, in our continued dialogue with participants during data collection, we did not hear any accounts of the morning report being any different than usual. We only observed morning reports in four different specialties. Including more specialties could have provided a broader picture and enhanced the transferability of our findings. In addition, the specific Danish context may limit transferability to other countries.

## Conclusion

Our study shows that there is a high degree of variation in the workplace learning opportunities that the morning report can provide. This variation related to both formal and informal learning opportunities. This indicates that teaching quality and potential learning outcomes may vary. Most of the formal learning opportunities comprised medical expert themes while non-medical themes were more often represented among the informal learning opportunities and thus not used to their full potential. Focusing more on the “hidden” social, ethical and organisational themes that are represented in informal learning opportunities might support the professional development of junior doctors. Eraut’s framework provided a valuable theoretical lens to grasp different types of teaching activities and learning opportunities that might be used or used better to make the most of the time doctors spend together during morning reports.

## Data Availability

The datasets generated and/or analysed during the current study are not publicly available due to ethical considerations, i.e., participants who were guaranteed anonymity, but are available from the corresponding author on reasonable request.

## Abbreviations

JD: Junior doctor
SP: Medical specialists and consultants
HD: Head of Department
IM: Internal medicine
S1, S2: Surgical departments
EM: Emergency department
